# Responsiveness and minimal important change of the Norwegian version of the Disabilities of the Arm, Shoulder and Hand questionnaire (DASH) in patients with subacromial pain syndrome

**DOI:** 10.1186/s12891-017-1616-z

**Published:** 2017-06-08

**Authors:** Tarjei Rysstad, Yngve Røe, Benjamin Haldorsen, Ida Svege, Liv Inger Strand

**Affiliations:** 1 Faculty of Health Sciences, Oslo and Akershus University College of Applied Sciences, Postboks 4, St Olavs Plass, Oslo, Norway; 2 Department of Physiotherapy, Martina Hansens Hospital, Bærum, Norway; 3 Norwegian Research Center for Active Rehabilitation, Department of Orthopaedics, Oslo University Hospital, Oslo, Norway; 4 Department of Global Public Health and Primary Care, University of Bergen, Bergen, Norway

**Keywords:** Dash, Responsiveness, Minimal important change, MIC, Cosmin, Physical therapy

## Abstract

**Background:**

The Disabilities of the Arm, Shoulder, and Hand questionnaire (DASH) is a valid and reliable patient-reported outcome measure (PROM). It was designed to measure physical disability and symptoms in patients with musculoskeletal disorders of the upper extremity, and is one of the most commonly used PROMs for patients with shoulder pain. The aim of this study was to examine responsiveness, the smallest detectable change (SDC) and the minimal important change (MIC) of the DASH, in line with international (COSMIN) recommendations.

**Methods:**

The study sample consisted of 50 patients with subacromial pain syndrome, undergoing physical therapy for 3–4 months. Responsiveness to change was examined by calculating area under the receiver operating characteristic curves (AUC) and testing a priori-formulated hypothesis regarding correlations with changes in other instruments that measuring the same construct. The SDC was calculated using a test re-test protocol, and the MIC was calculated by the anchor-based MIC distribution. MIC values for patients with low and high baseline scores were also calculated.

**Results:**

DASH appeared to be responsive, as it was able to distinguish patients who reported to be improved from those unchanged (AUC 0.77). All of the hypotheses were accepted. The SDC was 11.8, and the MIC was 4.4.

**Conclusion:**

This study shows that the Norwegian version of the DASH has good responsiveness to change and may thus be recommended to measure outcome in patients with shoulder pain in Norway.

## Background

Shoulder pain is common in the general population with a reported 1-year prevalence of 5–47% [1]. Subacromial pain syndrome is the most common diagnosis for patients with shoulder pain [2]. Numerous patient-reported outcome measures (PROMs) are available for the evaluation of shoulder disorders [3]. PROMs have become standard instruments in intervention studies, and are increasingly being used in clinical practice to capture patients’ self-reported function, disability and health [4]. The use of PROMs in clinical practice can lead to improved patient-clinician communication and enhance patient care and outcomes [5]. It is essential that PROMs demonstrate acceptable psychometric properties [6]. To obtain consensus of how measurement properties are defined and tested, an international Delphi process has been carried out to develop methodological guidelines, called the COnsensus-based Standards for the development of Measurement INstruments (COSMIN) [7, 8]. A prerequisite for a PROM to be used for evaluation in both clinical practice and research, is the instrument’s responsiveness [9]. Responsiveness is defined as “the ability of an instrument to detect change over time in the construct to be measured”, and is considered an aspect of validity [7]. To interpret change scores of a PROM two benchmarks are required: the smallest detectable change (SDC) and the minimal important change (MIC), which the COSMIN group defines respectively as “the smallest change that can be detected by the instrument, beyond measurement error” and “the smallest change in the construct to be measured which patients perceive as important” [9].

The DASH is a region specific PROM, and is according to Roy et al. [10] the most commonly used questionnaire for assessing shoulder function. It was developed to measure physical disability and symptoms of single or multiple disorders in the upper limb region [11], and has been translated into several languages including Norwegian [12]. The DASH has shown acceptable reliability, validity and responsiveness across a number of shoulder pathologies [10, 13].

However, according to the COSMIN panel inadequate methods for examining responsiveness and MIC have been applied, such as effect size (ES), standardized response mean (SRM) and distribution-based approaches [14]. The COSMIN panel claims that these methods measures the magnitude of an effect, and not the instruments ability to detect change over time or the importance of the change [7, 9]. Roy et al. [10] pointed out the need to conduct more studies regarding the estimation of MIC in shoulder disability scales. Since the responsiveness and MIC of a PROM may vary by population and context [15], it is important to examine these measurement properties in different samples of patients.

To our knowledge, responsiveness of the DASH has not previously been examined in line with the COSMIN recommendations. Hence, the purpose of this study was to assess responsiveness, and to determine and compare the SDC and MIC of the Norwegian version of the questionnaire following these guidelines.

## Methods

### Study participants and procedure

This clinimetric study included patients with subacromial pain syndrome (SPS). The patients were recruited from an outpatient clinic for physical therapy and rehabilitation at X, from December 2007 to October 2010. The patients were referred to “usual physical therapy” in primary health care, partly financed by the public health insurance service. The inclusion criteria was a diagnosis of SPS given by an orthopaedic surgeon based on clinical findings and symptoms, including anterior-lateral shoulder pain worsening during elevation of the arm and overhead activities, positive isometric abduction and positive impingement sign [16]. Exclusion criteria were systematic disease or generalized pain, cardiac disease, symptoms of cervical spine disease or surgery in the affected shoulder within the last 6 months. Patients were also excluded if they were unable to read or speak Norwegian fluently. Ethical approval was obtained by the Norwegian Regional Committee for Ethics, and all the participants signed a written consent form.

The assessments for the present study were completed at three different time-points: T1 (baseline), T2 (1–2 weeks after baseline) and T3 (3–4 months follow-up). At T1 they completed the DASH, Shoulder Pain and Disability Index (SPADI), Short Form 36 Health Survey (SF-36), Numeric Pain Rating Scale (NPRS), and provided demographic data. Active range of motion (AROM) (shoulder abduction, flexion, and internal and external rotation) was measured with a goniometer by a physiotherapist. At T2, the patients only filled out the DASH and SPADI. After completed treatment (T3) they filled out DASH, SPADI, SF-36, and NPRS, and AROM was measured. Perceived recovery was reported by the patients on a 3-level ordinal scale. Mean DASH scores of T1 and T2 were calculated as a basis for measuring change.

### The Disabilities of the Arm, Shoulder and Hand questionnaire (DASH)

The DASH consists of a 30-item scale that contains 21 physical function items, five symptom items and four social role items. Each item has five response options concerning the patient’s symptom severity and function of the upper extremity in activity during the previous week. The scores range from 0 to 100, where 100 reflects the most severe disability. The DASH was considered incomplete if more than 10% (missing rule) of the items scores were missing. Missing items were imputed as the average of the remaining items [17]. A previous study based on the same sample of patients demonstrated acceptable test-retest reliability, internal consistency and construct validity of the Norwegian version of the DASH [18].

### Other assessment tools

*The Shoulder Pain and Disability Index (SPADI)* measures shoulder pain and disability, and contains 13 items in two domains: pain (five items) and disability (eight items). The total score ranges from 0 to 100, lower scores indicating less pain and disability [19]. According to systematic reviews, the SPADI has demonstrated high reliability [10, 20] and satisfactory validity in large groups of patients with shoulder pain [21], but its validity and reliability has been questioned in a recent review [22]. The Norwegian version of SPADI used in this study has shown adequate validity and reliability [23].

*The Short Form 36 Health Survey (SF-36)* measures health in eight domains: physical functioning (PF), role-physical (RP), bodily pain (BP), perception of general health (GH), energy and vitality (VT), social functioning (SF), role limitation due to emotional problems (RE), and mental health (MH). The results range from the worst outcome (0) to the best outcome (100). The SF-36 was used as a generic measure of health, recommended to supplement condition-specific measures in shoulder patients [24]. The scoring was carried out following the published guidelines [25]. The Norwegian version of SF-36 has demonstrated acceptable psychometric properties [26].

*Numeric Pain Rating Scale (NPRS)* captures the patient’s level of pain. It is an 11-point scale that is anchored on the left with “no pain”, and on the right with “worst pain possible”. The patients were asked to rate their current pain within the last 24 h. NPRS has shown good reliability and responsiveness, with reported MIC values varying from 1.1 to 2.17 in patients with shoulder pain [27, 28].

*Perceived recovery.* At the 3–4 months’ follow-up (T3) the patients were asked to rate on a 3-level ordinal scale whether their condition had improved, was unchanged or had deteriorated since baseline. The use of a change scale is common, while designs and the number of scoring alternatives may vary. In a recent review change scales were shown to be reliable and responsive [29], and excellent test-retest reliability was demonstrated in patients with musculoskeletal disorders [30], although validity has been questioned [31, 32].

### Assessment of responsiveness

Both criterion and construct approaches were used to examine responsiveness [9]. As a criterion approach, the perceived recovery after treatment was used as an anchor (gold standard) for important changes in DASH scores. A dichotomous variable (the ‘improved’ versus ‘unchanged‘) from the measure of change (perceived recovery) was used to examine the discriminate ability of change scores from the questionnaires, using the receiver operating curves (ROC) method. The area under the ROC curve (AUC) was used as an indicator of responsiveness. AUC is a measure of the instrument’s ability to discriminate between two groups according to an external gold standard (here: perceived recovery). For sufficient responsiveness, an AUC over 0.70 is recommended [9].

The construct approach includes a priori hypotheses of expected associations between scores of DASH and other assessment tools that measure more or less the same construct (NPRS, SPADI, SF-36 and AROM). The strength of the positive correlation coefficients were interpreted according to Munro [33]: little, if any (.00–.25), low (.26–.49), moderate (.50–0.69), high (.70–0.89) and very high correlation (.90–1.00). Negative correlations were interpreted in a similar way. We used criteria described by de Boer et al. [34], which rates responsiveness as high if less than 25% of the hypotheses are refuted, moderate if 25–50% are refuted and poor if more than 50% are refuted. The hypotheses tested are explained in detail and presented in Table 1.

**Table 1:** A priori hypotheses to assess DASH responsiveness in patients with subacromial pain syndrome (*n* = 50)

No.	Hypotheses and rationales for the hypotheses	
1	The correlation between change scores of DASH and NPRS is moderate (*r* = 0.50–0.69). Disability and pain are different, but related constructs as shown in previous studies [54, 55].	Yes
2	The correlation between change scores of DASH and SPADI is high (*r* = 0.70–0.89). Both questionnaires assess disability in similar shoulder pain conditions, and high correlation coefficients have been shown in previous studies [13, 56].	Yes
3	The correlation between change scores of DASH and the subdomain SPADI function is higher than between change scores of DASH and the subdomain SPADI pain. This hypothesis is based on previous studies showing that DASH has a lower emphasis on pain than on function and disability [13, 57].	Yes
4	The correlation between change scores of DASH and the SF-36 subdomain Physical functioning (PF) is higher than between change scores of DASH and the SF-36 subdomain Bodily pain (BP). This hypothesis has the same rationale as hypothesis no. 3.	Yes
5	The correlation between change scores of DASH and AROM abduction is moderately and negatively correlated (*r* = − .50–0.69). This hypothesis is based on findings in previous studies [46, 58].	Yes
6	The correlation between change scores of DASH and AROM of abduction is higher than the correlation between change scores of DASH and other AROM movements. Abduction is a typical impairment in sub-acromial pain syndrome [59].	Yes
Number of accepted hypotheses (%)		6 (100)

*DASH* disabilities of the arm, shoulder and hand questionnaire, *NPRS* numeric pain rating scale, *SPADI* shoulder pain and disability index, *SF-36* The Short Form 36 Health Survey (SF-36), *AROM* active range of motion

### Measurement error by Smallest Detectable Change (SDC)

The patients did not receive any treatment between T1 and T2. These two measurement points were used to determine measurement error. Measurement error can be expressed by the standard error of measurement (SEM) and the SDC. The COSMIN-group defines SEM as the standard deviation (SD) around a single measurement [9], and it was calculated as the square root of the within-subject total variance of an ANOVA analysis [35]. The intra-class correlation coefficient (ICC) was computed using a two-way mixed effects model for total agreement. The SDC was calculated as 1.96 × √2 × SEM. If change is above this value in individual patients one can be 95% confident that it is not caused by measurement error [36].

### Minimal Important Change (MIC)

To explore interpretability of change scores, the SDC was compared to the MIC. To distinguish clinically important change from measurement error, the MIC should be greater than the SDC. The MIC was determined by using visual anchor-based MIC distribution based on the ROC method [37]. The perceived recovery was used as an anchor, dichotomized as ‘improved’ and ‘unchanged’. The subgroup ‘deteriorated’ was not included in the ROC curve analysis. The sensitivity and 1-specificity values from the ‘improved’ and ‘unchanged’ group were plotted on the y- and x-axis to distinguish the two groups. To define the MIC for the DASH a ROC cut-off point was detected, by finding the maximized value of both sensitivity and 1-specificity [37]. The anchor was considered acceptable if a minimum correlation of 0.5 was found between the change scores of the DASH and the anchor [38].

In recent studies the MIC of PROMs has been found to vary depending on the baseline scores [39, 40], and MIC was therefore also calculated for subgroups depending on the median of the baseline scores.

### Data analysis

The data was assumed to be normally distributed if there was no or minimal difference between the mean and median value, confirmed by histograms, by Q-Q plot and by the Shapiro-Wilk test. Pearson and Spearman correlation coefficients were used as appropriate depending on fulfilment of normality criteria. Floor and ceiling effects were considered to be present if the lowest or highest possible score was achieved by more than 15% of the patients [6]. The analysis was performed using the software IBM SPSS version 22 for Mac.

## Results

Ninety-four patients met the inclusion criteria, while 29 (31.0%) were unwilling or unable to participate, and two were excluded because of generalized pain. Of the 63 subjects, 13 dropped out before follow-up assessment after treatment (between baseline test and 3–4 months follow-up). The final study population consisted of 22 women and 28 men, with a mean age of 54.4 years (Table 2). The mean treatment length was 15.8 weeks. Table 3 presents the patient characteristic according to the perceived recovery in question.

**Table 2:** Demographic data of patients with subacromial pain syndrome

	Follow-up	Drop-outs
Number	50	13
Gender		
Female (%)	22 (44)	8 (61.5)
Male (%)	28 (56)	5 (38.5)
Age (SD)	54.4 (12.9)	48.8 (12.5)
Affected side		
Left (%)	26 (52)	4 (30.8)
Right (%)	14 (28)	5 (38.5)
Both (%)	10 (20)	4 (30.8)
Symptom duration (months)		
Mean (SD)	44.3 (72.8)	55.7 (72.5)
Working/student full time (%)	23 (46)	6 (46.2)
Sick listed 100% (%)	6 (12)	3 (23.1)
Partial sick listed (%)	5 (10)	3 (23.1)
Retired (%)	13 (26)	1 (7.7)
Receiving disability benefit (%)	2 (4)	0 (0)
Unemployed (%)	1 (2)	0 (0)

**Table 3:** Characteristics of the improved, unchanged and deteriorated patients at 3–4 months follow-up according to the anchor (*n* = 50)

	Improved^a^	Unchanged^a^	Deteriorated^a^
Number (%)	30 (60)	13 (26)	7 (14)
Gender (female/male) (%)	46.7/53.3	23.1/76.9	71.4/28.6
Age	53.7 ± 11.7	58.5 ± 15.0	50.1 ± 13.6
DASH Baseline	27.4 ± 12.1	29.7 ± 16.9	28.0 ± 14.3
DASH Follow-up	16.8 ± 13.3	30.6 ± 21.5	38.8 ± 13.3
DASH Change score	10.6 ± 11.7	- 0.9 ± 10.1	- 10.8 ± 8.8
SPADI Baseline	31.9 ± 11.7	40.8 ± 20.1	41.6 ± 19.0
SPADI Follow-up	18.6 ± 15.6	36.1 ± 20.2	54.5 ± 30.0
SPADI Change score	13.3 ± 14.7	4.7 ± 4.7	- 12.9 ± 16.3
NPRS Baseline	3.7 ± 1.9	3.9 ± 2.0	4.6 ± 1.5
NPRS Follow-up	2.5 ± 1.9	3.5 ± 2.3	6.6 ± 2.1
NPRS Change score	1.2 ± 2.0	0.5 ± 1.0	- 2.0 ± 2.2

*DASH* disabilities of the arm, shoulder and hand questionnaire, *SPADI* shoulder pain and disability index, *NPRS* numeric pain rating scale

^a^Data are means ± standard deviations unless denoted otherwise

At baseline, two of the DASH items (0.13%) were identified as missing. Five items were missing (0.33%) at T3. All of the completed DASH questionnaires had at least 27 of the 30 items answered (missing rule), and the missing items were imputed as the average of the remaining items.

### Responsiveness

**Fig. 1:**
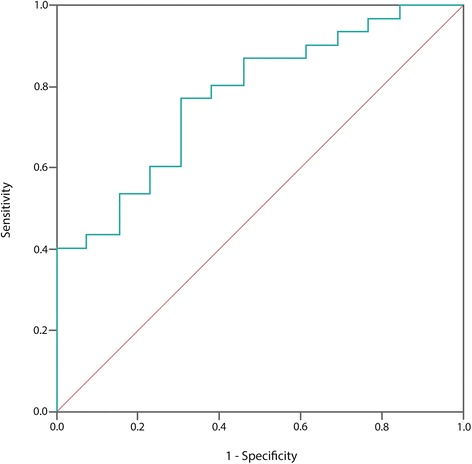
Receiver operating characteristic (ROC) curve for the change scores of DASH

Figure 1 presents the ROC curves generated for the DASH. Based on the anchor (perceived recovery), a total of 30 patients reported “improvement” and 13 “unchanged” (Table 2). The AUC of the DASH was 0.77 (95% CI: 0.63, 0.92).

Table 4 displays correlations coefficients between change scores for the instruments. All hypotheses regarding the DASH were confirmed, and are presented in Table 1.

**Table 4:** Pearson (*r*) or Spearman (*r* ^*s*^) correlation between the change scores of the DASH and other measurement tools (*n* = 50)

	DASH
Assessment tools	(0–100)
NPRS (0–10)	*r* ^*s*^ = 0.69
SPADI (0–100)	*r* = 0.82
SPADI Function (0–100)	*r* = 0.82
SPADI Pain (0–100)	*r* = 0.77
SF-36 Physical functioning (PF) (0–100)	*r* ^*s*^ = −0.61
SF-36 Physical limitations (RP) (0–100)	*r* ^*s*^ = −0.60
SF-36 Bodily pain (BP) (0–100)	*r* ^*s*^ = −0.53
SF-36 General health perception (GH) (0–100)	*r* ^*s*^ = −0.17
SF-36 Vitality (VT) (0–100)	*r* ^*s*^ = −0.30
SF-36 Limitation in social activities (SF) (0–100)	*r* ^*s*^ = −0.33
SF-36 Emotional problems (RE) (0–100)	*r* ^*s*^ = −0.46
SF-36 Mental health (MH) (0–100)	*r* ^*s*^ = −0.44
AROM Abduction	*r* ^*s*^ = −0.61
AROM Flexion	*r* ^*s*^ = −0.41
AROM Medial rotation	*r* ^*s*^ = −0.36
AROM Lateral rotation	*r* ^*s*^ = −0.26
Perceived recovery (anchor)	*r* ^*s*^ = −0.61

*DASH* disabilities of the arm, shoulder and hand questionnaire, *NPRS* numeric pain rating scale, *SPADI* shoulder pain and disability index, *SF-36* The Short Form 36 Health Survey (SF-36), *AROM* active range of motion

**Fig. 2:**
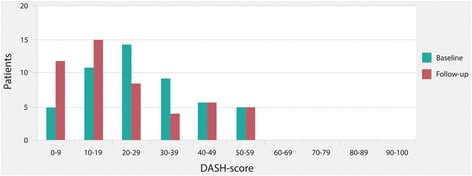
Score distribution of the DASH before and after the physical therapy. Higher values indicate greater disability

No floor or ceiling effects were observed for baseline or follow-up scores. The score distribution of the DASH is presented in Fig. 2.

### Smallest detectable change

The mean time period between T1 and T2 was 7.4 days (SD, 2.1). The ICC of the DASH was found to be 0.91. The SEM was 4.3, and the SDC was found to be 11.8.

### Minimal important change

Patients who reported deterioration on the perceived recovery (anchor) were excluded from the calculation of MIC. The anchor was considered appropriate since the correlation of both instruments with the anchor was higher than 0.5 (Table 4).

**Fig. 3:**
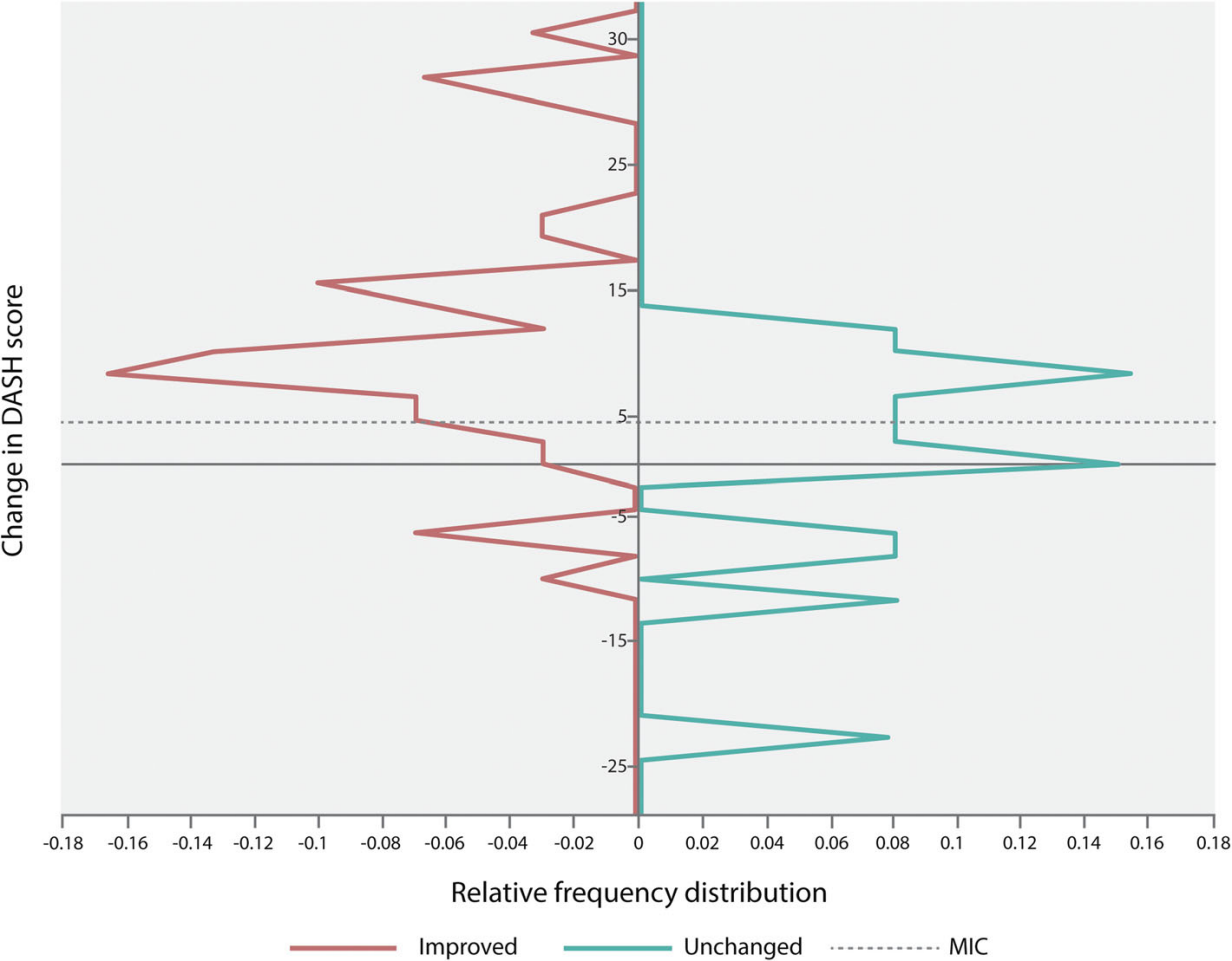
Anchor-based MIC distribution of the DASH with indication of the ROC cut-off point, after dichotomizing the patients in groups of improved and unchanged. MIC 4.4. Sensitivity at this point was 0.77 and specificity was 0.69

The MIC for the total change scores of DASH was 4.4, with a sensitivity of 0.77 and specificity of 0.69 (Table 5). Figure 3 illustrates the anchor-based distribution of the change scores for the improved and unchanged group, and the MIC value. Using the MIC as a cut-off point, 23.4% of the patients who were “improved” according to the anchor, had a lower change score and were considered false negatives. 30.8% of the patients, who were “unchanged” according to the anchor, had a higher change score and were considered false positives.

**Table 5:** Minimal important changes of the DASH (*n* = 50)

		Total sample			Low baseline scores					High baseline scores		
		MIC	SN	SP		MIC	SN	SP		MIC	SN	SP
DASH		4.4	0.77	0.69		4.4	0.73	0.71		7.7	0.73	0.83

Median DASH score = 26.4

*DASH* disabilities of the arm, shoulder and hand questionnaire, *MIC* minimal important change, *SN* sensitivity, *SP* specificity

MIC values for low and high baseline scores was respectively 4.4 and 7.7. The median DASH score was 26.4. Twenty-two patients (15 improved, 7 unchanged) had low DASH baseline scores (i.e. DASH ≤26.4). Twenty-one patients (15 improved, 6 unchanged) had high DASH baseline scores (DASH > 26.4). The MIC data are presented in Table 5.

## Discussion

The DASH is often used in research and clinical practice to evaluate disability and the effect of interventions for shoulder disorders. Therefore, it is important to evaluate its ability to capture change, and especially the smallest change score that patients perceive as important.

The study of responsiveness was conducted following all requirements of the COSMIN-checklist [41]: (1) Reporting the percentage of missing items, (2) describing how missing items were handled, (3) appropriate sample size. The total sample size included in the analysis, was within the range described as good (*n* = 50–99), except for the subgroup analysis and the calculation of the MIC which was poor (*n* < 30). (4) A longitudinal design with three measurements was used, (5) the time interval was stated, (6) the intervention was described, and (7) the proportion of patients that changed (i.e. improved, unchanged and deteriorated) was described. Regarding the hypotheses testing, (8) the hypotheses were formulated a priori, (9, 10) the expected direction and magnitude of correlations of the change scores were defined, (11, 12) the comparator instruments and its measurement properties were described. (13, 14, 16) The design, method and statistics are considered appropriate, (15) the criterion for change (anchor) can be considered to be a reasonable gold standard according to its correlation with the DASH (>0.50), (17) both correlation between the change scores and ROC curve were calculated, and (18) sensitivity and specificity for the patients who were ‘improved’ and ‘unchanged’ was determined.

For evaluating the responsiveness, both a criterion and a construct approach was conducted according to the COSMIN recommendations [9]. Using the criterion approach, this study found evidence for good responsiveness of DASH (AUC = 0.77, CI: 0.63, 0.92). This indicates that the questionnaire was able to distinguish patients who reported to have improved, from those who remained unchanged. The AUC-value found in the present study is similar to the findings reported in recent studies. In patients with a variety of shoulder disorders, Lundquist et al. [42] found an AUC of 0.76 (0.62–0.90). Michener et al. [43] found the AUC to be 0.79 (0.69–0.89) in patients with shoulder impingement. Similar to our findings, no floor or ceiling effect was reported in these studies, or in studies that included patients with upper-limb disorders [44, 45].

According to the construct approach, a total of 6 hypotheses were a priori formulated regarding the relationships between changes on the DASH and changes on related measures. None of the hypotheses were refuted. DASH showed little to moderate correlation with impairment measures, as is consistent with findings in other studies [46, 47].

We found a MIC value of 4.4 for the total DASH data. This implies that a change of five on the scale is likely to be considered important by individual patients. However, measurement error by SDC was found to be 11.8 which is a larger value than all calculated MIC values. This means that individual patients may considered a MIC value to be an important change. However, as it cannot be distinguished from measurement error it is not statistically significant [48]. Measurement error has to be taken into consideration when interpreting change scores. We should both be confident that the change in DASH is not simply due to measurement error and that it is sufficiently high to be important to individual patients. Hence, a change value must be over 11.8 for the DASH to be considered as an important change. In the present study 11 patients had a change value above 11.8, although 30 patients were considered improved according to the anchor.

Additionally, we found a higher MIC (7.7) for the patients with high baseline scores (Table 5). This supports previous findings that patients with higher baseline scores needs greater changes to be considered as important [37, 40]. MIC can also be expressed as percentage of baseline values [40]. When calculating the MIC values in our study as percentage, the MIC for the total DASH is 15.8% (4.4 of 27.9), and for the subgroups with low and high baseline scores, 25.9% (4.4 of 17) and 19.6% (7.7 of 39.2), respectively.

The SDC value found in our study is comparable with SDC values reported by others [36, 49, 50]. The MIC value (4.4) in the present study is, however, much lower as compared to results found in the literature. Gummesson et al. [51] found a MIC of 10 and van Kampen et al. [36] a MIC of 12.4. Beaton et al. [49] studied heterogeneous shoulder patients and compared different approaches for determine the MIC. Their results ranged from 3.9 to 15. These differing findings indicate how dependent the MIC is on the anchor. The anchor used in this study was a perceived recovery recorded on a 3-level ordinal scale, similar to what was used in a recent study [47] on responsiveness of the DASH. Using a 7- or 15-point Likert scale could have been better suited for this study, resulting in a more refined dichotomising between the subgroups. Nevertheless, such anchors have been criticized for not being capable of correlating functional change across varying lengths of time [52]. The anchor used in this present study can be questioned due to the large overlap between the distributions of the improved and unchanged group of patients illustrated in Fig. 3 according to the MIC value. However, in this study the anchor correlated above 0.5 with the change score of the DASH, and is then proposed to be a valid anchor according to a recent study [40]. Since there is no clear agreement of which method is the best to examine MIC, other approaches could have resulted in different MIC values. In addition, both the anchor-based approach and the COSMIN-group’s definitions of MIC and SDC used in this study, leads to a clear distinction between these two concepts [9, 53].

The present study is, to our knowledge, the first study to explicitly follow the COSMIN panel recommendation regarding examination of responsiveness of the DASH. However, there are some limitations as to how the findings should be interpreted. First, the COSMIN-checklist was developed to improve the selection of measurement instruments and how to evaluate these measurement properties. However, the checklist has not yet been tested for its reliability, and according to the authors it needs further refinement [41]. Second, the total sample size of this study is moderate (*n* = 50). Although it is within the COSMIN recommendation for examination of responsiveness, the sample size of the unchanged subgroup (*n* = 13) used for the analyses was small. Third, an ordinal scale with more scoring options might have been preferred.

## Conclusions

In conclusion, the Norwegian version of the DASH was found to be satisfactory responsive when applied on patients with subacromial pain. Based on the SDC and MIC, a change value on the DASH must be above 11.8 to be considered as an important change that is not due to measurement error.

## Abbreviations

AROM: Active range of motion
AUC: Area under the ROC curve
COSMIN: COnsensus-based Standards for the selection of health Measurement Instruments
DASH: The disabilities of the arm, shoulder and hand questionnaire
MIC: Minimal important change
NPRS: Numeric pain rating scale
PROM: Patient reported outcome measure
ROC: Receiver operating characteristic
SD: Standard deviation
SDC: Smallest detectable change
SEM: Standard error of measurement
SF-36: Short Form 36 Health Survey
SPADI: Shoulder pain and disability index
SPS: Subacromial pain syndrome
